# Supplementary material for: Full-scale scaffold model of the human hippocampus CA1 area
Source: Nat Comput Sci. 2023 Mar 23;3(3):264–76. doi: 10.1038/s43588-023-00417-2 (PMC10766517; doi:10.1038/s43588-023-00417-2)
Supplement: Supplementary file 1 — Supplementary Figures 1-6, Supplementary Tables 1-3 [file 43588_2023_417_MOESM1_ESM.pdf]

nature computational science

Resource https://doi.org/10.1038/s43588-023-00417-2

Full-scale scaffold model of the human hippocampus CA1 area

In the format provided by the authors and unedited

TABLE OF CONTENTS:

Supplementary Figures 3

Supplementary Tables 7

Supplementary Figure index

Supplementary Figure 1. Probability clouds of interneurons. 2
Supplementary Figure 2. Indegree and outdegree distribution of the null model. 2
Supplementary Figure 3 Network dependency on parameters changes. 3
Supplementary Figure 4 Full-scale network simulation. 4
Supplementary Figure 5 Single neurons activity. 5
Supplementary Figure 6 Co-simulation framework. 6

Supplementary Table index

Supplementary Table 1. Morphological parameters of probability clouds adopted to build the model. 6
Supplementary Table 2. Synaptic probabilities. 7
Supplementary Table 3. Numerical distribution of CA1 neurons in the model 7

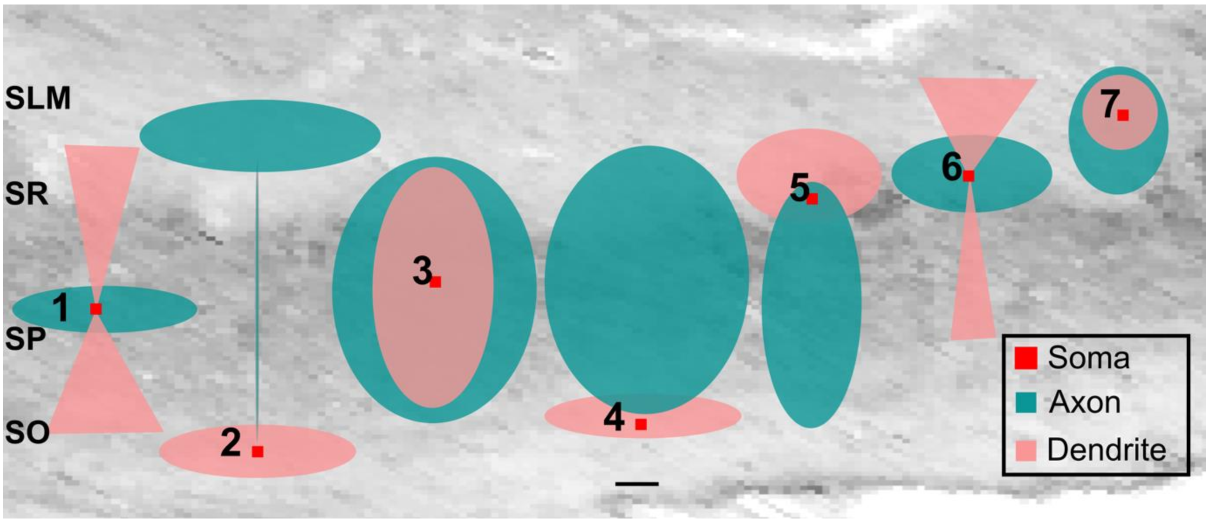

Supplementary Figure 1. Probability clouds of interneurons. Probability clouds for the classes of morphologies that have been generated to represent the 7 types of interneurons adopted in the present model. Red squares indicate the preferential position of the cell soma within the CA1 layers while green and pink shapes represent axonal and dendritic probability clouds. Morphological shapes of modeled interneurons are depicted on the original Nissl-Stained image of CA1 human hippocampus obtained from the BigBrain database. Note that layers (SLM,SR,SP,SO) are indicatively represented to suggest the subdivision in layer. 1. Perisomatic-like. 2. OLM-like. 3. IVI-like. 4. Trilaminar-like. 5. SCA-like. 6. PPA-like. 7. NGF-like

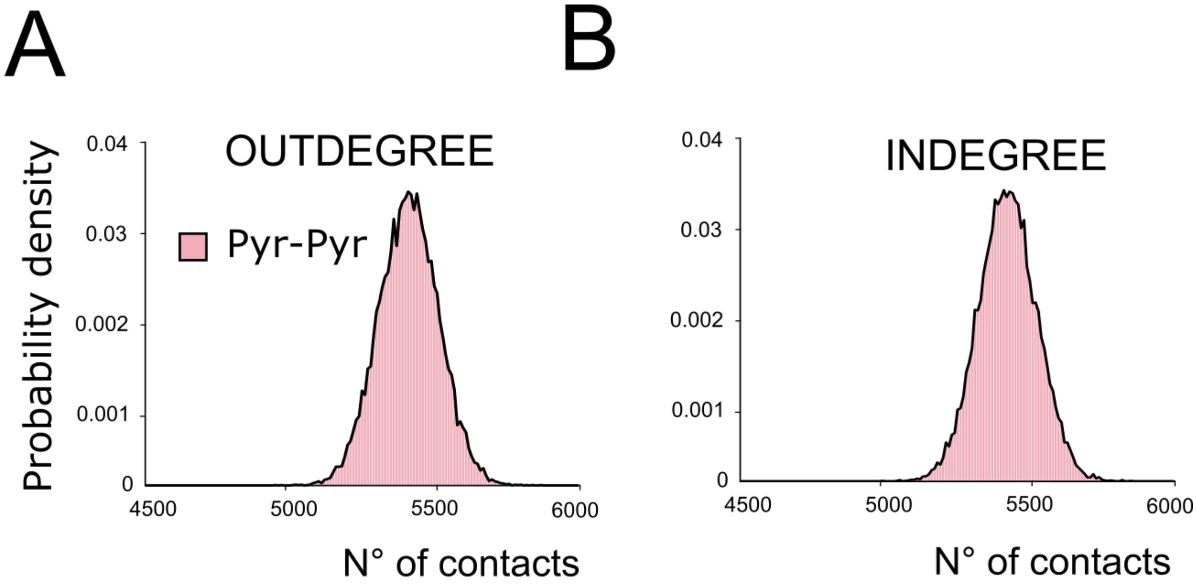

Supplementary Figure 2. Indegree and outdegree distribution of the null model. **A.** Outdegree probability density distribution obtained by connecting 100000 pyramidal cells sampled from the CA1 cell placement with all the rest of the pyramidal neurons in the CA1. Cells have been randomly connected with an exponential decay connectivity algorithm (Giacopelli et al 2021) with parameters obtained from the fitting of the probability density of distances generated by the model (Fig 7C main text). *Top.* Histogram shows the distribution of outdegree with a prominent peak at 5400 contacts and a very limited half-width (150 contacts). *Bottom.* The histogram shows an enlarged view of the same outdegree distribution shown on the top. **B.** Indegree probability obtained with the same

method described in A shows a Gaussian distribution with peak at around 5400 contacts with half-width of 150 contacts.

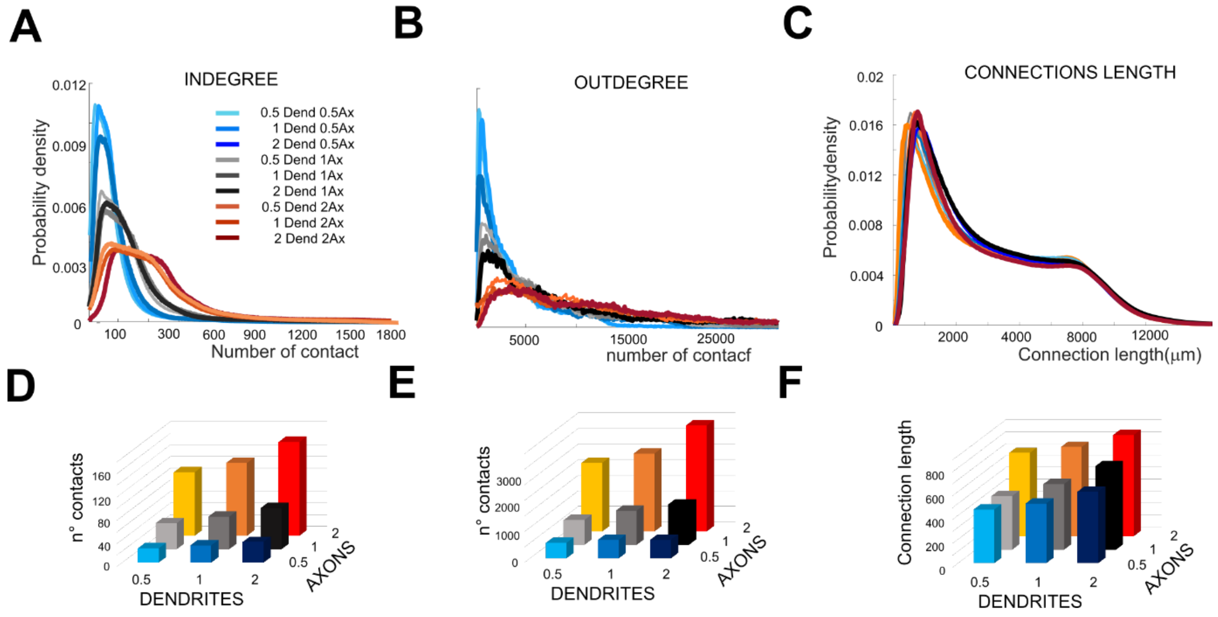

Supplementary Figure 3. Network dependency on parameters changes. The effects of parameterization have been evaluated on an excitatory network by changing axon and dendrites of Pyramidal cells and indegree, outdegrees and connection length have been estimated. Axons have been halved and doubled in their diameters whereas dendrites have been halved and doubled in both their height and radius. The connectivity algorithm has been repeatedly launched to calculate the 9 possible network configurations on a subpopulation of 200,000 pyramidal cells randomly selected and uniformly distributed within the CA1 and whose axons were intersected with all the PCs dendrites. The major impact of changing geometrical parameters was on the total number of connections, and especially in the case of doubling axons (+1500% compared to the default network). **A.** The indegree probability distribution has been obtained by connecting a subset of 200,000 pyramidal cells with all pyramidal cells in the modeled CA1. The PCs morphologies have been changed by generating axons with double and half diameter and dendrites with double and half height as well as double and half diameter cones. Color code represents traces obtained by modeling axons of the same size (blue 0.5 axon; gray 1 axon; red 2 axons). Color intensity represents traces obtained with the same dendritic size (from light for half dendrite to dark for twice dendrite). Curves with same axons size show similar shape indicating that PCs axons has the most prominent effect on network configuration. Note the lower small number of contacts compared to full network. **B** Similarly to A colored traces represent the outdegree distribution obtained with different network configurations. Also in this case, the main effects on the shape of the distribution is determined by axonal size. **C**. The connection length probability densities obtained in different network configurations show very limited effects on the curve’s shapes. **D-F** A prominent change induced to network architecture is the shift in the peak of the distribution that can be observed for all the tested parameters and represented as 3D histograms with color codes in accordance with curves shown in A-C.

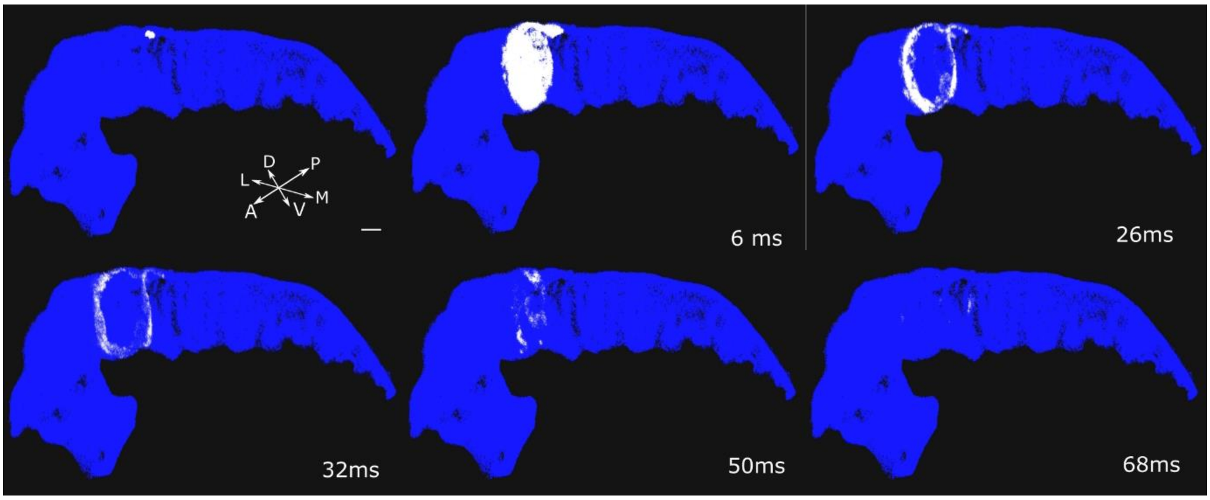

Supplementary Figure 4. Full-scale network simulation. Snapshots from a demo movie (Suppl. Movie 2) illustrating a simulation of the full network in which the activity was evoked by a single pulse stimulation delivered to about 6500 PCs in a 500 μm radius sphere near the CA2 region. Note that activity initially propagates in the transversal (medio-lateral) direction to subsequently spread longitudinally (antero-posterior) but differently from the purely excitatory network the activity spread is blocked by the action of the inhibitory network. Scale bar 2mm. Neuronal firing is coded by single neurons turning white (spiking) from blue (silent). Images generated with ViSimpl (https://vg-lab.es/visimpl/).

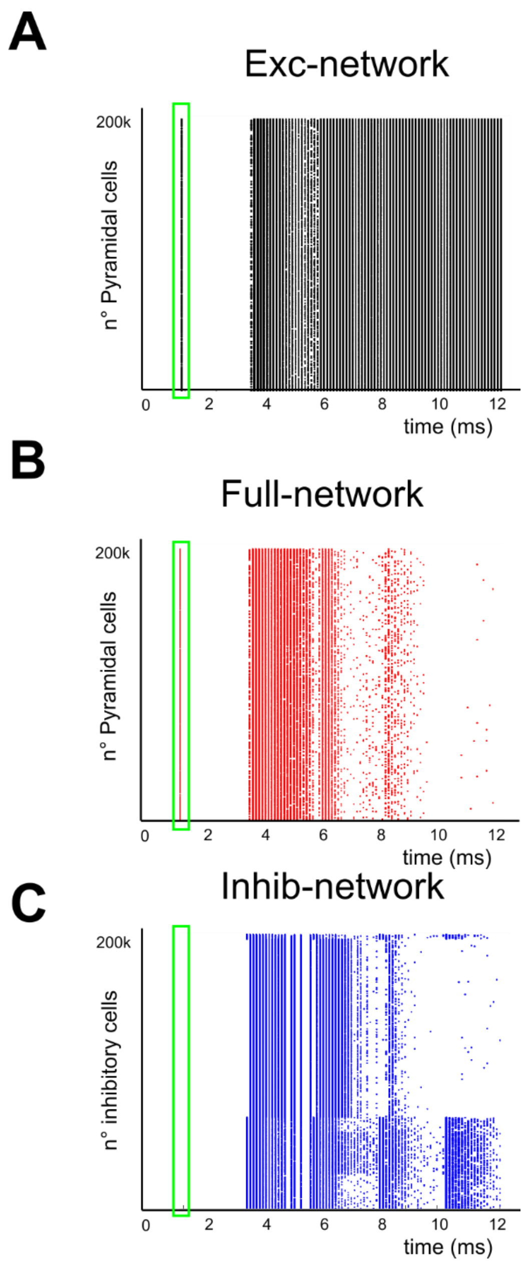

Supplementary Figure 5. Single neurons activity. **A-B** Raster plots of the firing activity of pyramidal cells activated in response to a single stimulus delivered to 6700 PCs in a spherical volume of 500μm radius in the case of a purely excitatory network (A) or in the presence of synaptic inhibition (B). The plots show only 200k of the 4.8M neurons which have been simulated. The stimulus is delivered at 1 ms (green box) **C** Raster plot shows the activity of inhibitory neurons during the simulation shown in B. Also in this case the plot shows 200k inhibitory interneurons among the 480k that have been included in the simulation. Note that since inhibitory network has been simulated with a unique neuronal and synaptic model (see Methods), the different firing activities are exclusively due to the network architectures.

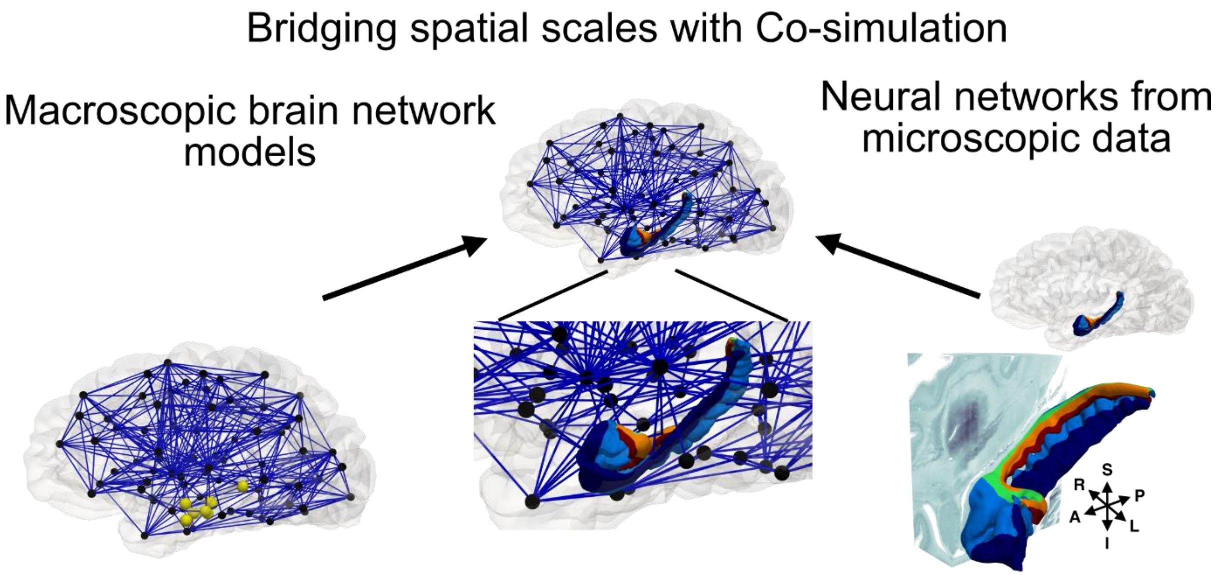

Supplementary Figure 6. Co-simulation framework Depiction of the Co-simulation workflow (work in progress). *Left:* Macroscopic brain network model with neural masses. The hippocampus is represented by 5 neural masses (yellow). *Right:* The hippocampus as a neural network model, constructed from high resolution microscopic data. *Middle:* The embedding of the detailed neural network model into the full brain model. Low resolution hippocampal neural masses have been replaced by the detailed neural network, maintaining the connection to the rest of the network. Co-simulation enables the interaction of micro- and macroscopic neural dynamics.

Neuronal class	Axon shape	Axon size (µm)	Dendrite shape	Dendrite size (µm)	Ref.
Pyramidal cell	Bending tube	Radius (150±15)	Cone (apical)	Height (300±30)	-Neuromorpho<br>-Mouselight<br>-Montero-Crespo et al 2020<br>-Benavides-Piccione et al 2020
				Radius (150±15)	
		Length (variable)	Cone (basal)	Height (500±100)	
				Radius (150±30)	
Perisomatic-like	Ellipsoid	Axis 1 (500±50)	Cone (apical)	Height (proportional to the soma positioning)	-Neuromorpho<br>-Pelkey et al 2017<br>-Bezaire and Soltesz 2013
		Axis 2 (500±50)		Radius (140±14)	
		Axis 2 (140±14)	Cone (basal)	Height (300±30)	
				Radius (250±25)	
OLM-like	Ellipsoid (projection)	Axis 1 (10±1)	Ellipsoid	Axis 1 (500±50)	-Neuromorpho<br>-Pelkey et al 2017<br>-Bezaire and Soltesz 2013
		Axis 2 (10±1)			
		Axis 3 (proportional to soma positioning)		Axis 2 (500±50)	
	Ellipsoid (plexus)	Axis 1 (600±60)			
		Axis 2 (600±60)		Axis 3 (150±15)	
		Axis 3 (250±25)			
IVY-like	Ellipsoid	Axis 1 (550±55)	Ellipsoid	Axis 1 (200±20)	-Neuromorpho<br>-Pelkey et al 2017<br>-Bezaire and Soltesz 2013
		Axis 2 (400±40)		Axis 2 (200±20)	
		Axis 3 (600±60)		Axis 3 (500±50)	
TRI-like	Ellipsoid	Axis 1 (500±50)	Ellipsoid	Axis 1 (400±40)	-Neuromorpho<br>-Pelkey et al 2017<br>-Bezaire and Soltesz 2013
		Axis 2 (500±50)		Axis 2 (400±40)	
		Axis 3 (Proportional to soma positioning)		Axis 3 (150±15)	
SCA-like	Ellipsoid	Axis 1 (200±20)	Ellipsoid	Axis 1 (300±30)	-Neuromorpho<br>-Pelkey et al 2017<br>-Bezaire and Soltesz 2013
		Axis 2 (200±20)		Axis 2 (300±30)	
		Axis 3 (Proportional to soma positioning)		Axis 3 (250±25)	
PPA-like	Ellipsoid	Axis 1 (300±30)	Cone (apical)	Height (proportional to the soma positioning)	-Neuromorpho<br>-Pelkey et al 2017<br>-Bezaire and Soltesz 2013<br>-Booker et al 2017
		Axis 2 (300±30)		Radius (100±10)	
		Axis 2 (250±25)	Cone (basal)	Height (400±40)	
				Radius (300±30)	
NGF-like	Ellipsoid	Axis 1 (200±20)	Ellipsoid	Axis 1 (150±15)	-Neuromorpho<br>-Pelkey et al 2017<br>-Bezaire and Soltesz 2013<br>-Price et al 2005
		Axis 2 (200±20)		Axis 2 (150±15)	
		Axis 3 (Proportional to soma positioning)		Axis 3 (150±15)	

Supplementary Table 1 Morphological parameters of probability clouds adopted to build the model. Note that the values provided in the table represent the peak ± half-width of the Gaussian distributions adopted to randomly sample values to assign geometrical parameters to ellipsoids and cones.

	PYRAMIDAL	Perisomatic-like	OLM-like	IVY-like	TRI-like	SCA-like	PPA-like	NGF-like
PYRAMIDAL	0.001272	0.00686	0.001417	0.00588	0.01302	0.005932	0.006596	0.00144
Perisomatic-like	0.001072	0.006858	0.001876333	0.006043	0.01326	0.001057	0.0044868	0.001382
OLM-like	0.00135	0	0.008255	0.00252	0.002992	0.00195	0	0
IVY-like	0.000933	0.00294041	0.001653571	0.005762	0.01306	0.0005491	0.0046174	0
TRI-like	0.002177	0	0.002141	0.00354	0.004053	0.001388	0	0.001965
SCA-like	0.0007354	0.00485285	0.001586	0.005296	0.01269	0.0006954	0.003997	0.001432
PPA-like	0.0008763	0.00347186	0.002104	0.008305	0.002836	0.001079	0.005401	0.001917
NGF-like	0	0	0.002001	0.005437	0.0098612	0.00187153	0.006543	0.001751

Supplementary Table 2 Synaptic probabilities. The probability of making contacts between two classes of neurons has been obtained from the public repositories Hippocampome (www.hippocampome.org) and it is referred to rodents. The number of putative contacts has been obtained by multiplying the total number of the presynaptic class (columns) per the total number of postsynaptic neurons (rows) per the synaptic probabilities.

EXCITATORY NEURONS		
PLACEMENT CLASS	NEURONAL CLASS	NUMBER
STRATUM PYRAMIDALIS	Pyramidal cells	4800000
INHIBITORY NEURONS		
PLACEMENT CLASS	NEURONAL CLASS	NUMBER
STRATUM ORIENS	OLM-like, Trilaminar-like	36278
STRATUM PYRAMIDALIS	Perisomatic-like	30448
STRATUM RADIATUM	IVY-like, SCA-like, PPA-like	186012
STRATUM LACUNOSUM MOLECOLARE	Neurogliaform-like	227262

Supplementary Table 3. Numerical distribution of CA1 neurons in the model. shows the numerical representativeness of Excitatory Neurons (Pyramidal cells) and Inhibitory neurons (OLM, Tri, Perisomatic, IVY, SCA, PPA, NG) subdivided based on the placement classes (SO, SP, SR, SLM).
