# Supplementary material for: Full-scale scaffold model of the human hippocampus CA1 area
Source: Nat Comput Sci. 2023 Mar 23;3(3):264–76. doi: 10.1038/s43588-023-00417-2 (PMC10766517; doi:10.1038/s43588-023-00417-2)
Supplement: Supplementary file 2 — Reporting Summary [file 43588_2023_417_MOESM2_ESM.pdf]

nature research

Corresponding author(s): DANIELA GANDOLFI, JONATHAN MAPELLI, MICHELE MIGLIORE

Last updated by author(s): Jan 31, 2023

Reporting Summary

Nature Research wishes to improve the reproducibility of the work that we publish. This form provides structure for consistency and transparency in reporting. For further information on Nature Research policies, see our Editorial Policies and the Editorial Policy Checklist.

Statistics

For all statistical analyses, confirm that the following items are present in the figure legend, table legend, main text, or Methods section.

n/a	Confirmed	
☐	☒	The exact sample size (n) for each experimental group/condition, given as a discrete number and unit of measurement
☐	☒	A statement on whether measurements were taken from distinct samples or whether the same sample was measured repeatedly
☒	☐	The statistical test(s) used AND whether they are one- or two-sided<br>*Only common tests should be described solely by name; describe more complex techniques in the Methods section.*
☒	☐	A description of all covariates tested
☒	☐	A description of any assumptions or corrections, such as tests of normality and adjustment for multiple comparisons
☐	☒	A full description of the statistical parameters including central tendency (e.g. means) or other basic estimates (e.g. regression coefficient) AND variation (e.g. standard deviation) or associated estimates of uncertainty (e.g. confidence intervals)
☒	☐	For null hypothesis testing, the test statistic (e.g. F, t, r) with confidence intervals, effect sizes, degrees of freedom and P value noted<br>*Give P values as exact values whenever suitable.*
☒	☐	For Bayesian analysis, information on the choice of priors and Markov chain Monte Carlo settings
☒	☐	For hierarchical and complex designs, identification of the appropriate level for tests and full reporting of outcomes
☒	☐	Estimates of effect sizes (e.g. Cohen's d, Pearson's r), indicating how they were calculated

Our web collection on statistics for biologists contains articles on many of the points above.

Software and code

Policy information about availability of computer code

Data collection	No software used to collect data
Data analysis	The codes allowing to implement the computational pipeline for a subset of Pyramidal cells and interneurons will be released as a Code Ocean capsule (https://doi.org/10.24433/CO.8325351.v1). Codes employed in this research have been developed though customized procedures mainly in the MATLAB (v2019b) environment.<br>The computational pipeline relies also on the following software/packages:<br>-Python 3.6.13<br>-Myavi 4.8.1<br>-NEST 3.0<br>-Excel (Windows 10)<br>-Jupyter Notebook 6.5.2<br>-Numpy 1.24.2<br>-Vsimpl 1.5.2

For manuscripts utilizing custom algorithms or software that are central to the research but not yet described in published literature, software must be made available to editors and reviewers. We strongly encourage code deposition in a community repository (e.g. GitHub). See the Nature Research guidelines for submitting code & software for further information.

Data

Policy information about availability of data

All manuscripts must include a data availability statement. This statement should provide the following information, where applicable:

- Accession codes, unique identifiers, or web links for publicly available datasets
- A list of figures that have associated raw data
- A description of any restrictions on data availability

Source data for Figure 3-5 are available with this manuscript. Source images are available from the BigBrain repository. The scaffold model resulting from the analysis of the images is available in the EBRAINS knowledge graph in the form of a text file with GIDs number and x,y,z coordinates that can be used to reproduce Figures 2 and 6. At the same link the connection pairs can be downloaded as a collection of multiple text files containing the GIDs of the presynaptic and postsynaptic neurons and a hdf5 file was generated to allocate each txt file as an hdf5 key.

Field-specific reporting

Please select the one below that is the best fit for your research. If you are not sure, read the appropriate sections before making your selection.

☒ Life sciences ☐ Behavioural & social sciences ☐ Ecological, evolutionary & environmental sciences

For a reference copy of the document with all sections, see nature.com/documents/nr-reporting-summary-flat.pdf

Life sciences study design

All studies must disclose on these points even when the disclosure is negative.

Sample size	No sample size was chosen since the full image database was considered to perform image analysis
Data exclusions	No data were excluded from our research
Replication	No replication has been performed since only a single image dataset was available
Randomization	Our study does not compare a positive and a control group, randomization is therefire not relevant to this study. A randomization procedure has been performed to connect neurons in an alternative fashion in respect to our approach.
Blinding	Our study does not include trials based on the concealment of group allocation of individuals

Reporting for specific materials, systems and methods

We require information from authors about some types of materials, experimental systems and methods used in many studies. Here, indicate whether each material, system or method listed is relevant to your study. If you are not sure if a list item applies to your research, read the appropriate section before selecting a response.

Materials & experimental systems

n/a	Involved in the study	
☒	☐	Antibodies
☒	☐	Eukaryotic cell lines
☒	☐	Palaeontology and archaeology
☒	☐	Animals and other organisms
☒	☐	Human research participants
☒	☐	Clinical data
☒	☐	Dual use research of concern

Methods

n/a	Involved in the study	
☒	☐	ChIP-seq
☒	☐	Flow cytometry
☒	☐	MRI-based neuroimaging
