# Supplementary material for: Full-scale scaffold model of the human hippocampus CA1 area
Source: Nat Comput Sci. 2023 Mar 23;3(3):264–76. doi: 10.1038/s43588-023-00417-2 (PMC10766517; doi:10.1038/s43588-023-00417-2)
Supplement: Supplementary file 10 — Densities values of voxels employed to generate plots of Fig. 3. [file 43588_2023_417_MOESM10_ESM.zip › Readme_Figure_3.rtf]

The 30_01_2023_Densities_Figures3.xlsx contains data required to generate the Figure and the plots in Figure 3.- The first tab of the Excel file (Voxels_D) has four columns with x,y,z coordinates (first 3 columns) of the center of the voxels, whereas the fourth column contains the density value of the associated voxels. - The second tab of the file (ML_axis) contains the values of the densities sampled in 25 (rows) voxels through three lines along the Pyramidal, Oriens and Radiatum layers repeated 10 times (columns).
