# Supplementary material for: Full-scale scaffold model of the human hippocampus CA1 area
Source: Nat Comput Sci. 2023 Mar 23;3(3):264–76. doi: 10.1038/s43588-023-00417-2 (PMC10766517; doi:10.1038/s43588-023-00417-2)
Supplement: Supplementary file 11 — 3D coordinates of axons and dendrites of 100 PCs used to generate images in Fig. 4. [file 43588_2023_417_MOESM11_ESM.zip › Readme_Figure_4.rtf]

The file PC_Axons.xlsx contains a tab with 3D coordinates to draw the first 100 axons of the 4.8 M Pyramidal cells.The second tab contains the GID of the neuron with a number indicating the number of points to be used for each axon. For example the first line of the GID tab has 141 	1. It means that the first axon has to be drawn with the first 141 3d coordinates of the coordinates tab file. The file PC_dendrites allow to generate the dendrites of the same first 100 PCs represented in PC_axons.
